# Percutaneous Occlusion of the Left Atrial Appendage with Thrombus Irresponsive to Antithrombotic Therapy

**DOI:** 10.3390/jcm10040726

**Published:** 2021-02-12

**Authors:** Krzysztof Kaczmarek, Iwona Cygankiewicz, Witold Streb, Rafal Plaksej, Piotr Jakubowski, Zbigniew Kalarus, Pawel Ptaszynski, Jerzy Krzysztof Wranicz, Anna Babicz-Sadowska, Agata Markiewicz, Marek Grygier

**Affiliations:** 1 Department of Electrocardiology, Medical University of Lodz, Pomorska Str 251, 92-213 Lodz, Poland; iwona.cygankiewicz@umed.lodz.pl (I.C.); pbjakubowski@wp.pl (P.J.); pawel.ptaszynski@umed.lodz.pl (P.P.); jerzy.wranicz@umed.lodz.pl (J.K.W.); 2 Silesian Center for Heart Disease, 1st Department of Cardiology and Angiology, 41-800 Zabrze, Poland; streb@wp.pl (W.S.); zbigniewkalarus@kalmet.com.pl (Z.K.); 3 Regional Center of Cardiology, Copper Medical Center 66 M. Skłodowska–Curie St., 59-300 Lubin, Poland; rplaksej@interia.pl (R.P.); sadowska.kardio@gmail.com (A.B.-S.); 4 1st Department of Cardiology, Poznan University of Medical Sciences, Dluga1/2, 61-848 Poznan, Poland; agatamarkiewicz@onet.eu (A.M.); marek.grygier@skpp.edu.pl (M.G.)

**Keywords:** left atrial appendage, thrombus, closure, anticoagulant, mechanical valve, percutaneous closure

## Abstract

We analyzed clinical experience with percutaneous closure of instances of left atrial appendage with thrombus (LAAT) irresponsive to antithrombotic therapy in patients treated in three high-volume cardiology centers. Clinical and procedural data regarding consecutive patients who underwent percutaneous left atrial appendage closure (PLAAC) due to LAAT were retrospectively analyzed. The study population consisted of 17 patients (11 men; 68 ± 14 years; CHA_2_DS_2_VASC 4.7 ± 1.9; HASBLED 3 (0–5)) with LAAT confirmed by transesophageal echocardiography, and included 5 patients with mechanical heart valves. Most of the patients (94.1%) received anticoagulation therapy before PLAAC. All LAATs were located in distal portions of the appendage and occupied less than 30% of its volume. Occluding-device implantation was successful in 17 patients; in one, a residual leak was disclosed. Appropriate positioning of occluders required more than 1 attempt in 6 individuals (35.3%); in 3 others (17.6%), the subjects’ devices had contact with thrombi. No procedural complications were noted. Midterm follow-up (median: 10 months) revealed no procedure-related complications or clinically diagnosed thromboembolism. Transesophageal echocardiography (TEE) performed after six months revealed device-related thrombus in one patient. We concluded that LAAT irresponsive to antithrombotic therapy might be effectively treated with PLAAC, even in patients with mechanical-valve prostheses.

## 1. Introduction

Left atrial thrombus formation is disclosed with transesophageal echocardiography in approximately 4% of patients with nonvalvular atrial fibrillation despite receiving appropriate therapy with vitamin K antagonists (VKA) or direct oral anticoagulants (DOAC) [1]. It is uncertain which therapeutic action should be undertaken in such a scenario. Standard clinical practice, however (not included in any recommendation), is to intensify anticoagulant therapy [2,3]. Invasive approaches including percutaneous endocardial or epicardial left atrial appendage (LAA) closure, and the surgical excision of LAA might be another alternative to cope with this problem [4,5]. Generally, in patients with nonvalvular atrial fibrillation and a high risk of embolic stroke (CHA_2_DS_2_–VASc of at least 2), percutaneous left atrial appendage closure (PLAAC) should be considered in the case of absolute contraindications for long-term oral anticoagulants (OAC) [6]. Additionally, if patients have an elevated bleeding risk score on OAC, refuse to take OAC, or are not compliant to this treatment, PLAAC may be considered as an alternative therapeutic option. Moreover, patients after LAA isolation during catheter ablation or those after stroke on OAC might be candidates for LAA closure [6]. Although LAA thrombus (LAAT) is generally considered a contraindication for any intervention in the left atrium [6], there were percutaneous LAAT closures reported as an ultima ratio treatment option [7,8,9]. Up to now, all published data, which are mostly case reports, cover fewer than 50 patients with percutaneously closed LAAT. The largest series of patients were reported by Tarantini et al. [5], who described 28 patients treated in 8 Italian centers, and by Bordignon et al. [10], who, in 2019, summarized a single-center German experience with 9 patients undergoing percutaneous LAAT closure. As this topic remains elusive and needs more evidence [6], we share the experiences of high-volume Polish centers with percutaneous closure of LAA with a thrombus inside. Additionally, for the first time, we present a subgroup of patients after open-heart surgery with a valve replacement in whom LAAT were jailed with endocardial occluders.

## 2. Material and Methods

### 2.1. Study Group

Consecutive patients who underwent percutaneous LAA closure (PLAAC) due to a thrombus in an appendage despite antithrombotic treatment were included in the study. Data were retrospectively collected from three high-volume centers. A decision of left atrium percutaneous closure despite thrombus was taken individually for each patient during heart-team (cardiologists, invasive cardiologists, and cardiac surgeons) meetings according to the routine elaborated in each center. Inclusion criteria to the study were: (1) A decision from the local heart team of PLAAC in the patient with LAAT and (2) subsequent commencement of an invasive procedure with the intention of PLAAC.

In our study population, there were 12 (70.6%) patients with typical indications for left atrial appendage closure (LAAC) according to “EHRA/EPCI expert consensus statement on catheter-based LAAO—an update” [6]. This group included patients with contraindications for long-term oral anticoagulants due to a history of bleeding (8 patients, 47%) and those with ineffective anticoagulation that resulted in stroke (4 patients, 23.5%). The remaining 5 (29.4%) patients had LAAT diagnosed during transesophageal echocardiography (TEE) performed before catheter ablation of left atrial arrhythmias. These patients were treated with anticoagulants prior to the ablation procedure as a standard way of care in patients with atrial arrhythmias.

Patient demography, past medical history, indications for PLAAC, course of procedures, and further follow-up were analyzed. Details regarding preprocedural preparation and technical aspects of PLAAC were examined. The data were directly obtained from physicians who took care of the patients. The diagnostic and therapeutic management of patients was left to the discretion of each center.

### 2.2. Diagnostic and Therapeutic Procedures

**Figure 1:**
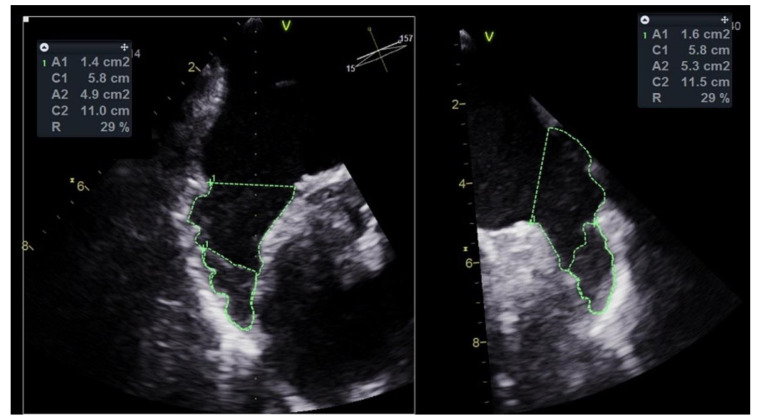
Measurement of percentage of left atrial appendage volume occupied by thrombus in transesophageal echocardiography (TEE) (Supplementary Video S1). A1 and A2, Areas 1 and 2; C1 and C2, Circumferences 1 and 2; R, ratio A1/A2.

Patients had TEE performed just before and during the PLAAC procedure, in which the thrombus was visualized in multiple views. Thrombus position in LAA was described as located in a proximal or distal portion of LAA. Additionally, the mean percentage of LAA volume covered by thrombus was approximated with a calculation of mean percentage of area covered by LAAT to the LAA area obtained from two TEE projections—30°–60° and 120°–150° (Figure 1). Anatomical characteristics of LAA included: (1) classification to one of four LAA shape variants (chicken wing, windsock, cauliflower and cactus); (2) measurement of the longest (D1) and shortest (D2) diameters of the LAA orifice in the landing-zone position; (3) measurement of LAA depth; and (4) calculation of the orifice eccentricity index (EI) according to an equation: EI = 1 − D2/D1 [11].

Procedural details that were analyzed were: (1) the maximal size of the implanted occluder; (2) mean oversizing of the implanted device, defined as the mean difference between the occluder diameter and orifice diameters (D1 and D2); (3) percentage mean oversizing, defined as the mean oversizing divided by the mean of longest and shortest orifice diameter; (4) the number of occluder positioning attempts; (5) device contact with thrombus during implantation; (6) mobilization of the thrombus into the left atrium; (7) deviation from center’s procedural routine applied in regular non-LAAT patients; (8) duration of TEE and PLAAC procedure (skin to skin). Procedural success, including device and technical success, and complications related to the PLAAC were defined according to the Munich consensus document [12].

### 2.3. Follow-Up

Follow-up visits were conducted according to the standard of care of each center, which in all cases included clinical evaluation after 6 weeks, 6 months, and 12 months, with TEE performed 6 months after the PLAAC procedure. Information about antiplatelet and/or anticoagulant drug therapy was collected from medical records at four crucial time-points: (1) on hospital admission for PLAAC, (2) during hospital stay before procedure, (3) on discharge, and (4) after 6 months of follow-up.

### 2.4. Statistical Analyses 

Statistical analysis was performed using Statistica software (ver. 13, StatSoft Inc., Tulsa, OK, USA). Continuous variables were tested for normality with the Shapiro–Wilk test and presented as mean ± standard deviation if normally distributed; otherwise, as median and range. Categorical variables are shown as numbers and percentages. The Student’s t- and Wilcoxon–Mann–Whitney tests were applied for between-group comparisons according to data distribution. The chi-squared test and its modification were used to compare categorical data. Values of *p* < 0.05 were considered statistically significant.

### 2.5. Ethical Approval 

The study was conducted according to Declaration of Helsinki and approved by the bioethical committee of the Medical University of Lodz, Poland (RNN/223/18/KE; 12.06.2018). Each patient gave written and informed consent before having the PLAAC procedure performed.

## 3. Results

### 3.1. Study Group’s Clinical Characteristics 

Clinical and imaging data (both echocardiographic and fluoroscopic) were complete and of sufficient quality to obtain all required information in all 17 (100%) cases (Table 1 and Table 2). The study group consisted of 17 patients (11 men; 64.7%) aged 34–91 (68.2 ± 13.9) years. All patients suffered from atrial tachyarrhythmias, most commonly atrial fibrillation. The overall risk of thromboembolic events (CHA_2_DS_2_VASC score—4.7 ± 1.9) and bleeding episodes (HASBLED score—3 (0–5)) were high.

Six patients had previous cardiothoracic surgery (35.3%). Of those, 5 (29.4%) were valve replacement, including 4 (23.5%) who received mechanical prosthesis, of which 3 (17.6%) were in aortic and 1 (5.9%) in mitral positions. The remaining one patient (5.9%) underwent surgical excision of LAA, but 2 years later, TEE revealed an LAA remnant with a thrombus inside (Supplementary Figure S1). Patients with mechanical-valve prosthesis (MVP) had similar demographic (age: 64.8 ± 3.9 vs. 69.3 ± 15.8, *p* = 0.4; male sex: 75% vs. 61.5%, *p* = 0.6) and clinical characteristics (CHA_2_DS_2_VASC: 5.0 ± 0.8 vs. 4.6 ± 2.1, *p* = 0.7; HASBLED: 3.3 ± 1.5 vs. 2.8 ± 1.4, *p* = 0.6; EF: 43.0 ± 13.9% vs. 44.0 ± 15.5%, *p* = 0.8) to those of other patients; however, statistical comparison was limited by a small sample size (4 vs. 13 patients). Detailed clinical characteristics of the study group are summarized in Table 1, and detailed data of each patient are shown in Supplementary Table S1.

### 3.2. Antithrombotic Treatment

Antithrombotic treatment was deemed optimal for all patients. A majority of the study group, 15 patients (88.2%), received oral anticoagulants; 10 (58.8%) of them were on VKA and 5 (29.4%) took DOAC. The 2 (11.8%) remaining individuals were treated atypically. The first was on enoxaparin due to a severe episode of intracerebral hemorrhage on DOAC. The second patient, a 91-year-old woman, received aspirin monotherapy (75 g daily) because all other treatments caused recurrent gastrointestinal bleeding that required blood transfusions. After LAAT diagnosis, the initial antithrombotic therapy was intensified in 12 patients (70.6%), which included the use of high doses of unfractionated heparin intravenously in 1 patient (5.9%), and a combination of anticoagulants (oral or heparins) and antiplatelets (aspirin or/and clopidogrel) in 11 individuals (64.1%). Nevertheless, none of these regimens was fully effective in any patient. However, in all seven patients (41.2%) in whom infusion of unfractionated heparin was administered for at least 7 days, the LAAT had reduced in size; therefore, they had LAAC procedures performed on uninterrupted antithrombotic therapy.

### 3.3. Echocardiographic Findings

All four anatomical variants of the LAA were revealed in the study group (Table 2). Cauliflower shape was the most common (7 patients; 41.7%). In one individual who had undergone open-chest surgery for LAA elimination due to LAAT despite adequate anticoagulation, a remnant of this anatomical structure was left. As the patient was scheduled for catheter ablation of left atrial arrhythmia, preprocedural TEE was performed and revealed LAAT. The LAA orifices had diameters of 22.5 ± 4.2 mm (longest) and 18.6 ± 2.5 mm (shortest), with an eccentricity index of 0.12 (0.05–0.45), and 34.0 ± 5.5 mm of depth. The thrombi occupied approximately one-fifth of the appendage (22.1 ± 5.6%) and were located in a distal portion of the LAA in all patients.

### 3.4. Percutaneous Closure of Left Atrial Appendage with Thrombus Procedure

All PLAACs were performed in intravenous conscious sedation, and catheters were inserted through the right femoral vein. Both fluoroscopy and TEE were used for intraprocedural imaging guidance. After trans-septal puncture, the target Activated Clotting Time (ACT) of more than 250 s was obtained in all patients. Maneuvers in the left atrium were performed gently, which meant changing wires in the left pulmonary vein and introducing catheters only to proximal parts of LAA. Auriculography, considered a typical step of PLAAC in all centers, was not performed in 12 (70.5%) patients due to the expected risk of thrombus mobilization, ischemic stroke, and/or systemic thromboembolization. Five patients recruited in one center underwent LAA angiographies with gentle hand contrast injections through pigtail catheters with no clinical signs of stroke or other thromboembolisms.

**Figure 2:**
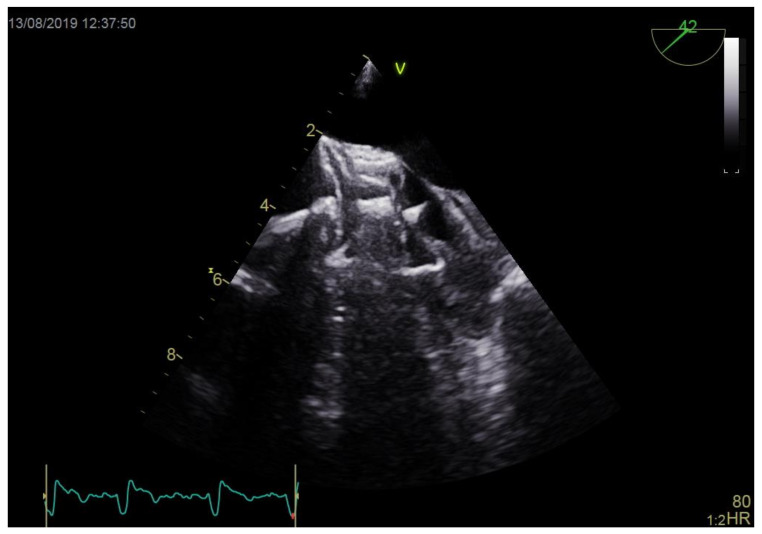
Occluder in contact with left atrial appendage thrombus.

Technical details of the occluding devices used in PLAAC procedures are summarized in Table 3. Devices were successfully positioned during the first attempt in 11 (64.7%) patients. Three patients (17.6%) had the device repositioned once, and 3 other patients had the device repositioned 3 times. Implanted occluders had diameters 6.3 ± 3.9 mm larger than the LAA orifice, which accounted for 15.2 ± 3.9% of mean percentage oversizing. Eventually, occluders were implanted in appropriate positions in all patients, jailing LAAT and passing stability tests. In one individual, a residual perioccluder leak of a > 5 mm jet was revealed after the device had been deployed, but due to the high eccentricity of the LAA ostium (EI = 0.45), no better position was available. During occluder positioning in 3 patients (17.6%), the devices had direct contact with thrombi (Figure 2, Supplementary Video S2). Nonetheless, in none of these cases was the clot mobilized or were clinical signs of intraprocedural thromboembolic events observed.

No center reported the use of cerebral protection devices; however, during 10 procedures, Sentinel systems (Claret Medical, Santa Rosa, CA, USA) were in operating rooms prepared to be used if needed. All implantations were finished with acute clinical success. No procedure-related or inhospital complications were reported in any of these patients (Table 3).

On discharge, patients most frequently received dual antiplatelet therapy (7 patients; 41.2%); less often, oral anticoagulants with one antiplatelet drug (5 patients; 29.4%); and rarely, triple therapy (3 patients; 17.6%). One of the two remaining patients had warfarin prescribed as monotherapy, and the second was prescribed 75 mg clopidogrel once daily.

### 3.5. Follow-Up

Median follow-up duration was 10 months (1–18). Eleven patients (64.7%) were observed for at least 6 months after LAAC. There were no midterm-procedure-related complications due to thromboembolic or hemorrhagic events. One patient died due to the progression of heart failure. Five of the 11 patients (45.4%) who had completed the half-year follow-up were on aspirin monotherapy. Three (17.6%) individuals were on a combination of VKA and aspirin, and the remaining two (11.8%) on dual therapy of DOAC with acetylsalicylic acid (ASA). One patient did not receive any anticoagulant or antiplatelet because she had gastrointestinal bleeding that required a blood transfusion while on ASA. Dual therapy with VKA and ASA was upheld in three patients (17.6%) with a history of mechanical-valve implantation. One patient initially treated with Dual antiplatelet therapy (DAPT) had thrombus on the occluder, which dissolved after therapy modification to dabigatran and clopidogrel.

## 4. Discussion

The main finding of our multicenter analysis is that the percutaneous closure of left atrial appendage thrombi that are irresponsive to standard anticoagulation might be feasible and safe with uneventful midterm follow-up in patients with nonvalvular atrial fibrillation and in those with prior mechanical-valve implantation. Additionally, the intraprocedural direct contact of the clot with an occluder and intraprocedural gentle LAA angiography did not lead to a clinically overt thromboembolic event.

Study groups in two previously published papers regarding the percutaneous closure of LAAT were significantly different, especially in terms of comorbidities and indications for PLAAC. Tarantini et al. [5] presented 28 individuals with nonvalvular atrial fibrillation who had been referred for PLAAC mainly due to major bleeding events on anticoagulants (53%) and so-called “malignant LAA” (29%), which was defined as LAA-persistent thrombus connected to thromboembolic events. On the other hand, Bordignon et al. [10] reported a very specific group of patients consisting of a high percentage (67%) of individuals in whom LAA thrombi were discovered after the electrical isolation of LAA (LAAI), which was performed as a part of an invasive treatment of atrial tachyarrhythmias. Our population that was similar to the one described by Tarantini et al. [5] included several patients with a history of major bleeding events (47%) and individuals in which an LAAT was found accidentally before arrhythmia catheter ablation. More importantly, our population included patients after surgical valve replacement. To the best of our knowledge, only one paper reported a single case of percutaneous LAAT closure in mechanical-valve recipients [13]. This notwithstanding, as thrombi in our patients were located in the LAA and irresponsive to anticoagulation therapy, the percutaneous implantation of LAA occluders was considered to be a unique treatment option. Technically, PLAAC procedures were similar to those in patients without artificial valves, but in individuals with mitral valve prosthesis, special attention was paid to not entrap catheters within mechanical-valve discs and to position occluders with no collision with the valve. Three patients with MVP completed midterm clinical and echocardiographic evaluations, which were uneventful. There is a lack of evidence for PLAAC in patients after MVP implantation because these patients were not included in either LAAC registries or clinical trials [14,15]. Recently, two reports were published and showed favorable results in patients undergoing percutaneous mitral valve procedures and concomitant PLAAC [16,17].

The optimal therapeutic approach for patients with LAAT is still debatable. Some authors argue that the intensification of anticoagulant therapy or changing VKA to DOACs is sufficient [2,3]. Contrastingly, there are examples of devastating failures of such an approach, which subsequently led to abandoning such treatment at one of the high-volume centers [9]. Moreover, due to a high bleeding risk, uptitration of anticoagulants or applying a combination of antiplatelet and anticoagulation therapy is not feasible in many patients, especially from a long-term perspective [18,19,20]. The majority (70%) of our patients had their pharmacotherapy previously modified with no success. Antithrombotic treatment directly prior to PLAAC procedures remains another important issue. Some of our patients had procedures performed on an infusion of unfractionated heparin in order to maintain a small thrombus size and prevent the possible formation of clots on endovascular tools. Unfortunately, neither the Italian nor German groups [5,10] reported this important periprocedural detail. Experience gathered from catheter ablations of atrial fibrillation showed that instrumentation of the left atrium can be relatively safely performed on uninterrupted anticoagulant therapy [21]. Moreover, there is no agreement regarding postprocedural treatment with anticoagulants and/or antiplatelets. Tarantini et al. [5] showed that over one-third (39.3%) of their patients on discharge received dual antiplatelet therapy, which was followed by oral anticoagulants (28%) and low-molecular-weight heparin (18%), while approximately 10% had combinations of antiplatelets and oral anticoagulants prescribed. After 6 months, similarly to the observations of other authors [5,10], approximately half of the patients (45%) in our group were only on aspirin, although many (45%) still remained on oral anticoagulants. There is a general consensus to prescribe aspirin monotherapy for half a year after an LAA closure procedure in typical conditions [6]. The discrepancy in our group was mainly influenced by patients with mechanical prosthesis in whom VKA therapy was mandatory. Two nonvalvular patients had DOAC with clopidogrel initiated, the first because a device-related thrombus had developed on DAPT, and the second due to a left atrial spontaneous echo contrast detected in TEE. Sedaghat et al. [22] reported on relatively frequent (more than 15%) clot formation on LAA occluders. DOAC initiation was documented to successfully resolve device-related thrombi, which was also confirmed in one of our patients.

Technical aspects of the procedure are of high importance mostly due to the considerable threat of intraprocedural thromboembolism. There was no assumption that any particular device was more suitable for percutaneous occlusion of LAA with a thrombus inside. The selection of the device was left to the discretion of the physician performing the procedure. All authors advise performing procedures in a gentle manner with as few maneuvers as possible within LAA [7,8,9,10]. There are suggestions that some of the procedure steps be omitted if possible, i.e., LAA angiography, occluder contact with thrombus, or device repositioning [5,10]. In our study group, LAA angiography was usually omitted, but in a few patients who had had it performed, no thrombus mobilization and no clinical signs of stroke were revealed. There are single reports showing that preimplantation auriculographies were performed [7], although generally, this part of the procedure is thought to be risky [5]. Considering our data and all published by others, there are globally 10 LAAT patients in whom occluders were repositioned during PLAAC, and in none was the thrombus mobilized, nor did stroke occur [5,10]. Direct contact between occluder and LAAT was observed in 17% of patients in our study population. In all of them, occluders were successfully deployed in the first attempt. Some authors advised the use of cerebral protection systems during PLAAC in LAAT patients, although such an approach has not been commonly adopted [5,23]. We are in favor of cerebral protection, especially in patients in whom there is a substantial risk of mobilization of the thrombus (i.e., deep occluder insertion, shallow LAA, or proximal thrombus). These circumstances probably do not happen so frequently because almost all patients reported in the literature had thrombi in distal parts of the LAA [5,10]. Similarly, in our group, LAA clots occupied 30% or less of LAA volume and were located in the distal half of LAA. In the case of larger thrombi, an additional option, successfully applied in 41% of our patients, was to try to reduce its size with extraordinary preprocedural intensive therapy using anticoagulants or antiplatelets. Recently, the use of isoproterenol for emptying echocontrast or sludge prior to LAAC was reported [24,25]. This method could be included in diagnostic and therapeutic approaches in patients with LAAT; however, more data, especially of its safety, are needed.

All authors report favorable data on PLAAC of LAAT from a periprocedural perspective and from midterm follow-ups. Therefore, considering our experience and data from two previous papers [5,10], a multicenter, international, and preferably randomized prospective study should be planned comparing the pharmacological therapy of LAAT irresponsive to optimal anticoagulation to the invasive closure of thrombi inside of LAA.

Important limitations of our analysis are the small sample of the study population and the retrospective data analysis. Additionally, as we did not perform preprocedural and postprocedural cerebral imaging, post-PLAAC silent embolic events cannot be excluded.** Due to the retrospective nature of the study, differences in diagnostics and therapy between the centers exist.

## 5. Conclusions

Left atrial appendage thrombus irresponsive to antithrombotic therapy might be effectively treated with percutaneous closure, including in patients with prior mechanical-valve implantations. Percutaneous left atrial appendage occluders may be safely implanted in LAAT patients even if difficult intraprocedural conditions are met, including direct contact between thrombus and occluding device.

## Figures and Tables

**Table 1:** Clinical characteristics of study population.

Clinical Characteristics	
Age, years	68.2 ± 13.9
Sex, male	11 (64.7%)
CHA_2_DS_2_VASC	4.7 ± 1.9
CHA_2_DS_2_VASC > 3	14 (82.4%)
HASBLED	3 (0–5)
HASBLED > 2	10 (58.8%)
Coronary artery disease	7 (41.2%)
Arterial hypertension	14 (82.4%)
Diabetes	7 (41.2%)
Renal failure	5 (29.4%)
Heart failure HFrEF HFmEF	9 (52.9%) 6 (35.3%) 3 (17.6%)
Left ventricular ejection fraction (%)	43.7 ± 14.7
Atrial fibrillation/flutter Paroxysmal Persistent Permanent	17 (100%) 3 (17.6%) 6 (35.3%) 8 (47.1%)
Previous stroke Ischemic Hemorrhagic	10 (58.8%) 8 (47.1%) 2 (11.8%)
Previous cardiac surgery Biological prosthesis Mechanical prosthesis LAA excision	6 (35.5%) 1 (5.9%) 4 (23.5%) 1 (5.9%)
Previous implanted pacemaker/ICD device	4 (23.5%)
Anticoagulant or antiplatelet treatment before LAA procedure: VKA DOAC ASA Enoxaparin	17 (100%) 10 (58.8%) 5 (29.4%) 1 (5.9%) 1 (5.9%)
Antithrombotic therapy intensification before LAA procedure:	
Indication for PLAAC Contraindication for OAC Stroke on OAC LAA thrombus on OAC/APT	12 (70.6%) 8 (47.1%) 4 (23.5%) 5 (29.4%)

HFrEF, heart failure with reduced ejection fraction; HF, heart failure with midrange ejection fraction; ICD, implantable cardioverter-defibrillator; VKA, Vitamin K antagonists; DOAC, direct oral anticoagulants; ASA, acetylsalicylic acid; OAC, oral anticoagulant; LAA, left atrial appendage; APT, antiplatelet therapy.

**Table 2:** Echocardiographic characteristics of study population.

Echocardiographic Parameters	
LAA anatomical type Cauliflower Windsock Chicken wing Cactus Undetermined (remnant of LAA)	7 (41.2%) 4 (23.5%) 3 (17.6%) 2 (11.8%) 1 (5.9%)
LAA orifice diameter (mm) Longest Shortest	22.5 ± 4.2 18.6 ± 2.5
LAA depth (mm)	34.0 ± 6.3
LAA orifice eccentricity index	0.12 (0.05–0.45)
LAA thrombus location (portion) ½ proximal ½ distal	0 (0.0%) 17 (100%)
Thrombus burden area within LAA/LAA area (%)	22.1 ± 5.6

LAA, left atrial appendage.

**Table 3:** Characteristics of percutaneous left atrial appendage closure procedures.

Cardiac rhythm during procedure Sinus rhythm Atrial fibrillation/flutter	2 (11.8%) 15 (88.2%)
Anesthesia Conscious sedation General anesthesia	17 (100%) 0 (0.0%)
Implanted device Amplatzer amulet Watchman Watchman FLX	13 (76.5%) 2 (11.8%) 2 (11.8%)
Device size (mm)	27 (18–34)
Device mean oversize (mm)	6.3 ± 1.9
Device mean oversize (%)	15.5 ± 3.9
Device deployment attempts 1 attempt >1 attempt	1 (1–4) 11 (64.7%) 6 (35.3%)
Auriculography	5 (29.4%)
Procedural complication	0 (0.0%)
LAAC success Device success Technical success Procedural success	17 (100%) 16 (94.1%) 16 (94.1%)
TEE duration (min)	46 (20–90)
Skin–skin procedure duration (min)	40 (30–80)

LAAC, left atrial appendage closure; LAA, left atrial appendage; TEE, transesophageal echocardiography.

## Data Availability

The majority of the data presented in this study are available in Supplementary Table S1. Further data presented in this study are available on request from the corresponding author.

## Supplementary Materials

The following are available online at https://www.mdpi.com/2077-0383/10/4/726/s1. Video S1: left atrial appendage thrombus, Video S2: contact between occluder and left atrial appendage thrombus during device insertion, Figure S1: left atrial appendage remnant with thrombus inside, Table S1: clinical and procedural details of each patient.
