# Supplementary material for: Percutaneous Occlusion of the Left Atrial Appendage with Thrombus Irresponsive to Antithrombotic Therapy
Source: J Clin Med. 2021 Feb 12;10(4):726. doi: 10.3390/jcm10040726 (PMC7918449; doi:10.3390/jcm10040726)
Supplement: Supplementary file 1 [file jcm-10-00726-s001.pdf]

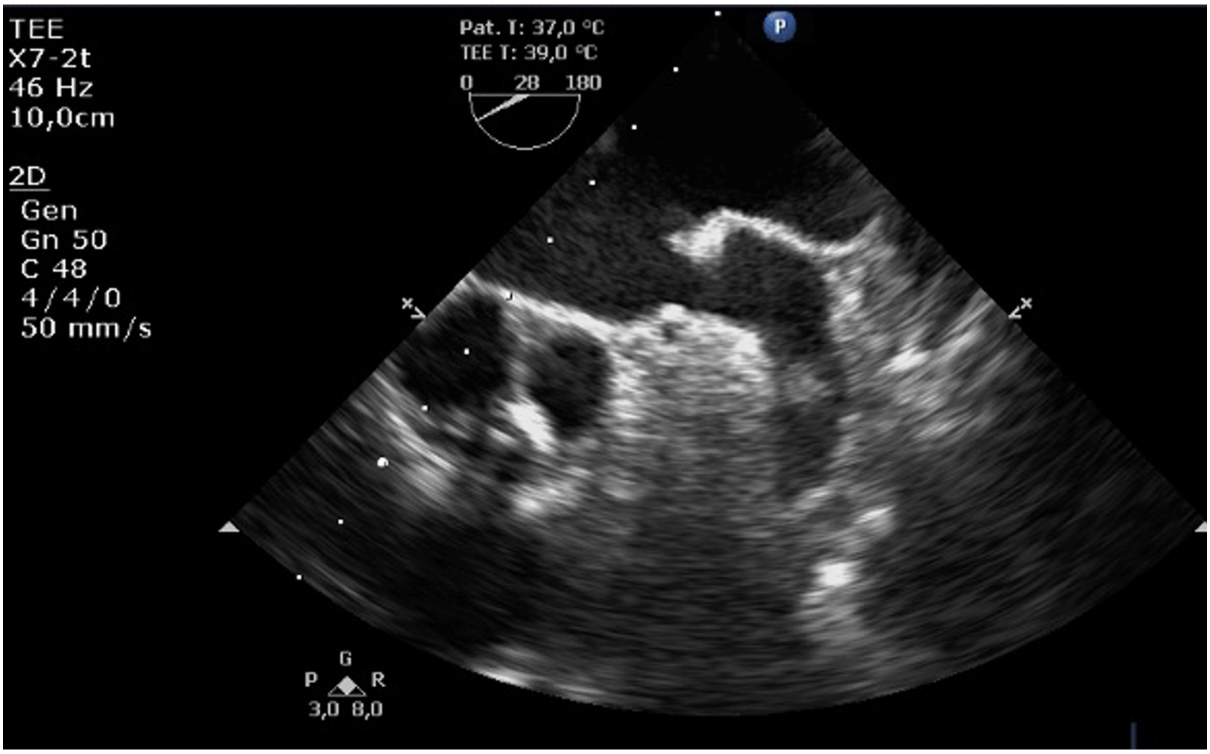

Figure S1. Left atrial appendage remnant with thrombus inside.

Table S1. Clinical and procedural details for each patient.

Patient	Age	Sex	EF	Indication for LAAC	Chronic optimal OAT	Preprocedural modification of AT (regimen)	Anatomical type of LAA	Size of LAA ostium [mm]	Thrombus/ LAA (%)	Occluding Device	Occluder Size	Oversize (%)	Procedural Time (skin-skin) [min]
1	34	F	62	Stoke on OAC	Warfarin	Yes*	Windsock	24x19	30	Amp.Amulet	28	15.1	60
2	84	M	42	GI Bleeding	Dabigatran	No	Windsock	20x18	20	Amp.Amulet	25	15.8	40
3	66	M	15	LAAT	Warfarin	Yes*,**	Chicken-wing	19x18	14	Amp.Amulet	25	17.6	40
4	91	F	59	GI Bleeding	ASA	Yes***	Cauliflower	32x19	18	Amp.Amulet	34	16.7	80
5	49	M	32	LAAT	Warfarin	Yes***	Cactus	18x16	15	Amp.Amulet	22	14.7	45
6#	69	F	57	Stroke on OAC	Warfarin	Yes**	Windsock	25x22	20	Amp.Amulet	31	16.0	45
7	63	F	32	GI Bleeding	Warfarin	Yes**	Cauliflower	18x17	18	Amp.Amulet	22	12.9	30
8	56	M	35	LAAT	Dabigatran	Yes*, ****,*****	Remnant	17x15	25	Amp.Amulet	18	6.3	30
9	80	M	40	Hemorrhagic Stroke	Apixaban	No	Cauliflower	24x21	29	Watchman Flx	31	18.9	40
10	66	F	60	GI Bleeding	Warfarin	Yes*	Cauliflower	26x22	26	Watchman Flx	31	14.6	30
11	77	M	60	Urogenital Bleeding	Apixaban	Yes*, *****	Chicken-wing	21x17	25	Watchman	27	21.1	30
12	78	M	55	Hemorrhagic Stroke	LMWH	No	Cauliflower	25x22	24	Watchman	27	7.4	30
13#	62	M	50	LAAT	Warfarin	No	Cactus	21x18	24	Amp.Amulet	25	14.1	30

14	79	F	55	GI Bleeding	Rivaroxaban	No	Cauliflower	18x16	31	Amp.Amulet	22	14.7	50
15	78	M	25	Stroke on OAC	Warfarin	Yes*,****	Chicken-wing	25x23	20	Amp.Amulet	31	14.6	60
16#	61	M	40	LAAT	Warfarin	Yes**,******	Windsock	29x16	12	Amp.Amulet	31	18.9	65
17#	67	M	25	Stroke on OAC	Warfarin	Yes***, ******	Cauliflower	22x18	24	Amp.Amulet	28	20.0	50

#- patients with mechanical valve prosthesis. Regimens of preprocedural AT therapies: *- Dabigatran+ASA; ** - UFH+SAPT; ***- UFH; ****- VKA+ASA; *****- Enoxaparine+SAPT; ******- VKA+DAPT. EF – ejection fraction, LAAC – left atrial appendage closure, OAC – oral anticoagulants, OAT – oral antithrombotic therapy, AT – antithrombotic therapy; LAA – left atrial appendage, LAAT - left atrial appendage thrombus, GI – gastrointestinal, UFH – unfractionated heparin, ASA – acetylsalicylic acid, SAPT – single antiplatelet, DAPT – dual antiplatelet therapy; Amp.- Amplatzer.
